# Hospital Contracts and Prices

**Published:** 1910-04-09

**Authors:** 


### Special Departmental Article.

### HOSPITAL CONTRACTS AND PRICES.

**Contributions and questions on matters affecting this department of an institution are invited.**

The skilled supervision, or lack of skilled supervision, of the expenditure of a hospital is one of the factors, probably the chief factor, in determining its economical administration or otherwise. Hospital administrators should, and the majority of them do, make a point of dealing in the cheapest market consistent with quality, and they should lay down for themselves a golden rule not to order goods until they know the cost. Indiscriminate ordering is responsible for a good deal of heavy expenditure. Nothing, or next to nothing, should be bought without a quotation. The method of obtaining a quotation differs with different goods. Sometimes a contract covering a period is advisable, sometimes a quotation for a definite supply. In considering this question we naturally turn to the first sub-head in the Uniform System of Accounts, viz. Provisions, which is the most serious item of hospital expenditure and one in which proper methods of purchase and excellent quality of goods are particularly important.

### Times for Contracts.

Provisions should, as a rule, be purchased by contract covering a period, but it is wise to vary the period and also the time of making the contract. There are times in the year when certain articles of food are at their cheapest. Tradesmen are human and they are more optimistic when goods are plentiful and cheap than when they are scarce and dear, and purchasers are more likely to make favourable contracts during times of plenty than in those of scarcity. Excellent prices, from the purchaser's point of view, can usually be obtained if contracts are arranged as follows:—

Milk and butter annually in March for April 1, or June for July 1.

Fish annually in May for June 1.

Chickens annually in June, fowls in September.

Jams annually as soon as the new season's supplies are ready.

Groceries half-yearly in March and September.

Bread and flour, meat, bacon, cheese, etc., quarterly.

Potatoes and other vegetables, and also eggs, are the only provision items for which we do not advocate fixed contracts, experience having proved that it is cheaper and more advantageous as regards freshness and quality to obtain weekly quotations from large dealers. Purchasing potatoes in large quantities when at their cheapest and pitting them has been frequently tried, but this is not an economical method when the labour involved and waste caused by extra consumption and by perishing are taken into account. Purchasing eggs at their cheapest and preserving them has been advocated, but this involves labour, large storage accommodation, and serious risk of loss.

### Complete Contracts.

The tender forms for provisions should be of the most comprehensive character. Every item of groceries, different kinds of bacon, cheese, etc., should be included in the forms, and every joint in use noted in the meat schedule. Exception may be taken to this in consequence of the labour involved in examining the tenders, but it pays in practice. In large hospitals a price for fore and hind quarters of beef, and for carcases of mutton and lamb, should be included, in addition to a schedule of joints. Rounds of beef should be specified without bone and free from worthless udder fat and flanks. Sirloins and ribs should not be cut longer than twelve inches, and the former should be free from suet, so also should loins of mutton, and necks of mutton should be trimmed of outside fat. Shins and legs of beef if purchased with bones should be London cut, fair, and in sets. Suet should be the best beef kidney. The contract for fish should specify that it should be delivered cleaned, ready for cooking, and without heads, and, in case of cod fish, without the thin pieces of flap which turn brown when cooked and look unappetising. Milk should be specified pure and to give not less than 10 per cent. of cream and 3.5 per cent. of fat. The bread should be of the same quality for both patients and household, and should be excellent. To prevent waste by the rejection of crusts, the loaves for patients should be long and weigh eight pounds each, so that there are only two crusts in each eight pounds. Bread should be delivered cold within 12 hours after being baked, and should be weighed in bulk on reception and only the actual weight paid for.

### Weekly Payments.

An important factor in the economical purchase of provisions is the insertion in the conditions of contract that the account shall be paid weekly. The knowledge that at the end of each week a cheque will be received for the week's supplies is an inducement to tradesmen to tender, and the competition being keen a minimum of profit is the sult, much to the benefit of the institution. The preparation of the weekly account for provisions is also an excellent check upon extravagance, as it enables comparisons to be made of every provision sub-head with the previous week and with the corresponding week of the previous year. The weekly cost per head can also be compared.

### Form of Contract.

The following general conditions of contract have been found useful, and can be commended to Hospital Committees who are not satisfied with their present tender forms:—

| | |
|---|---|
| I, or we, | , hereby Tender to supply at the above Institution during the months commencing on the day of , 19 , the Articles as per Schedule, at such times and in such quantities as may be required, in accordance with, and agreeably to, the Conditions and Schedule mentioned herein. |
| | Dated this day of , 19 . Signature. Address. |
| | General Conditions of Contract. |
| Quality of Supplies. | 1. The Supplies shall be of the best quality, and in all respects equal to samples. |
| Mode of Order and Delivery. | 2. All goods must be supplied to a Written Order, and in no other way, and must be delivered, at the Contractor's expense, in such quantities and at such times as may be specified in the demands, and must be accompanied by an invoice. |
| Inspection. | 3. All Supplies shall be subject to the approval of the Committee, or such Officer as they may appoint, and the Contractor shall, when required, remove and exchange at his own expense such quantities as may be rejected. |
| Purchases in Default. | 4. If the Contractor shall fail to deliver the Supplies demanded at the specified time and place, or to replace those rejected as above mentioned, the Committee, or such Officer as they may appoint, shall be at liberty to purchase Supplies to replace those which may be deficient, and the Contractor shall defray all costs, charges, and expenses attendant upon such purchase, and all payments under his Contract shall be suspended until he shall have paid for such purchase. |
| Mode of Payment. | 5. At the expiration of each WEEK the Contractor shall submit his account for Supplies, and after each account has been examined and found correct it shall be paid by the Secretary. |
| Termination. | 6. In the event of non-compliance with these Conditions, the Committee shall have full power to terminate this Contract by giving one week's notice. |
| Sub-letting. | 7. This Contract shall not be sub-let without the written authority of the Committee. |
| Power to Accept any Part of Tender. | 8. The Committee do not bind themselves to accept the lowest or any Tender, and they reserve to themselves the power of accepting any part of a Tender. |

If a hospital does not possess cold storage and is using chilled meat, it is a good arrangement to insert a clause in the contract that the meat shall, if required, be delivered free from frost, and at such an hour that it can, as a rule, be cooked the same day. If quarters of beef or carcases of mutton are purchased, a clause stating that it shall be cut up on hospital premises by the supplying firm after being weighed is also an advantage.

### Accepting Tenders.

Too much care cannot be taken in selecting contractors for hospital supplies. The majority of committees safeguard themselves by stating both in the advertisement for tenders and in the conditions included in the tender form that "they do not bind themselves to accept the lowest or any tender, and they reserve to themselves the power of accepting any part of a tender." Firms occasionally quote prices for goods at figures which render it absolutely impossible for them to comply with the first condition of the contract, namely, that the supplies shall be of the best quality. They fully intend to ignore this condition, and send to the hospital something which they consider to be good enough. It is not to the advantage of the hospital to give an unfair price for any article, because if the hospital official who receives the goods is conscientious and experienced there is constant warfare between the hospital and the contractor respecting the quality of the supplies. Rejections are frequent, delays arise, and inconvenience is suffered both by patients and household. The committee, in considering the tenders, are wise if they reject any quotations which are obviously unfair, although, on the other hand, care must be taken that, in exercising their discretion, they are not doing both the hospital and the contractor an injustice. It is essential that members of the Contracts Committee should be fair judges of the value of goods, for great discrimination is needed to arrive at a just decision.

*(To be continued.)*

